# Efficacy of allogeneic tendon material coracoclavicular ligament reconstruction combined with Kirschner wire and titanium alloy hook plate material fixation in the treatment of acromioclavicular joint dislocation

**DOI:** 10.3389/fbioe.2024.1388905

**Published:** 2024-04-08

**Authors:** Bing Du, Yibo Xu, Zhao Li, Shuai Ji, Cheng Ren, Ming Li, Kun Zhang, Teng Ma

**Affiliations:** ^1^ Honghui Hospital, Xi’an Jiaotong University, Xi’an, Shaanxi, China; ^2^ Medical College of Yan’an University, Yan’an, Shaanxi, China

**Keywords:** joint dislocation, coracoclavicular, ligament allograft tendon, hook plate, kirschner wire

## Abstract

**Objective:**

To compare the effects of allogeneic tendon coracoclavicular ligament reconstruction combined with Kirschner wire fixation and clavicular hook plate fixation on early postoperative pain, postoperative shoulder joint function score and shoulder joint mobility in patients with acromioclavicular joint dislocation.

**Methods:**

From January 2020 to January 2023, 43 patients with acromioclavicular joint dislocation admitted to Xi ‘an Honghui Hospital were included. Among them, 24 patients were treated with the clavicular hook plate technique (Hook Plate,HP) group, and 19 patients were treated with allogeneic tendon coracoclavicular ligament reconstruction combined with the Kirschner wire technique (Allogeneic Tendon, AT) group. The Constant-Murley score of shoulder joint function 6 months after operation, postoperative shoulder joint activity, preoperative and postoperative pain, operation time, intraoperative blood loss and complications were compared between the two groups.

**Results:**

All 43 patients were followed up for an average of 9.7 (9–12) months. The intraoperative blood loss in the allogeneic tendon group was less than in the hook plate group. The Constant-Murley shoulder function score was higher than that in the hook plate group 6 months after the operation. The abduction and lifting activity was greater than that in the hook plate group. The visual analogue scale scores at 3 days and 14 days after operation were lower than those in the hook plate group. The difference was statistically significant (*p* < 0.001). There was 1 case (5.3%) of exudation around the Kirschner needle track in the allogeneic tendon reconstruction group, and 5 cases (20.8%) of complications in the hook plate group, including 1 case of internal fixation stimulation, 2 cases of acromion impingement syndrome, 1 case of acromioclavicular joint osteoarthritis, and 1 case of shoulder joint stiffness. The complication rate of the allogeneic tendon group was lower than that of the hook plate group.

**Conclusion:**

The clinical efficacy of allogeneic tendon coracoclavicular ligament reconstruction combined with Kirschner wire fixation in treating acromioclavicular joint dislocation (Rockwood type III-V) is better than hook plate internal fixation. The patients have less early postoperative pain and better recovery of shoulder joint function and shoulder joint mobility.

## 1 Background

Acromioclavicular dislocation is a common shoulder trauma disease, accounting for about 9% of shoulder diseases (Nordin et al., 2020). Among them, Rockwood type III and above should be treated with surgical fixation (Markel et al., 2017; Cook and Krul, 2018). The focus of surgical treatment is to reconstruct the acromioclavicular ligament and coracoclavicular ligament. The common fixation methods include hook plate fixation, Kirschner wire fixation, tendon reconstruction, loop plate fixation, etc (Batın et al., 2016; Arirachakaran et al., 2017; Lee et al., 2023; Ruiz et al., 2023). The clavicular hook plate is a special plate with a transverse groove on one side. It can achieve vertical and horizontal stability through the fixation of the plate and screw. Because of the low failure rate of internal fixation and the relatively early functional exercise of the shoulder joint, it is widely used in clinical practice. Although the hook plate is fixed in the clinical treatment of acromioclavicular joint dislocation, it has achieved good clinical treatment effect, but there is still some literature points out some shortcomings of the fixation technology, such as subacromial osteolysis, acromioclavicular arthritis and rotator cuff injury complications (Chandrasenan et al., 2007; Hoffler and Karas, 2010; Lin et al., 2014). Therefore, the steel plate should be removed as soon as possible after ligament repair. The TightRope plate coracoclavicular ligament reconstruction method is to fix two titanium buttons on the clavicle and under the coracoid process. Four FiberWire coils connect the titanium buttons through the bone tunnel to achieve the effect of coracoclavicular ligament reconstruction. Some scholars believe that this fixation method can only be fixed in the vertical direction, and its horizontal stability is difficult to maintain so that dynamic backward displacement will occur (Minkus et al., 2017). A mechanical experiment on the treatment of acromioclavicular joint dislocation with allogeneic tendon showed that the strength of allogeneic tendon material was sufficient for the reconstruction of coracoclavicular ligament, and its biomechanical strength was excellent (Beitzel et al., 2012). We believe that the treatment of acromioclavicular joint dislocation should follow its original biomechanical conditions as much as possible. In the past, the application of titanium alloy material hook plate accounted for the highest proportion, and the application of other materials was less, and the immediate fixation effect after operation was good. However, the long-term complications caused by the rigid structural properties of hook plate titanium alloy materials cannot be ignored (Li et al., 2024). Therefore, it is of great significance to find a suitable material and surgical method to replace the original hook plate fixation scheme to improve the postoperative complications of patients. Tendon coracoclavicular ligament reconstruction includes autologous tendon reconstruction, allogeneic tendon reconstruction and artificial tendon ligament reconstruction. The advantage of allogeneic tendon transplantation compared with autologous tendon reconstruction is that it avoids the process of autologous tendon extraction and has less surgical trauma to patients. Some scholars have applied allogeneic tendon materials and synthetic materials to the clinical treatment of acromioclavicular joint dislocation. The results show that allogeneic tendon materials can provide better clinical and imaging results (Fauci et al., 2013). The process of reconstruction is to bypass the free graft from the base of the coracoid process, drill the bone tunnel on the clavicle, and fix the transplanted tendon on the borehole (Han et al., 2021). This ligament reconstruction method is close to the original biomechanical structure of the coracoclavicular ligament, making the acromioclavicular joint less anterior and posterior translation (Mazzocca et al., 2006). The traditional method of Kirschner wire fixation is helpful to repair the acromioclavicular ligament and coracoclavicular ligament. However, once the Kirschner wire is displaced, other complications may occur, in addition to the loss of stability of the acromioclavicular joint. Therefore, Kirschner wire fixation can be used as an auxiliary fixation (Lateur et al., 2016).

In this study, the coracoclavicular and acromioclavicular ligament reconstruction was realized using allogeneic tendon coracoclavicular ligament reconstruction combined with the Kirschner wire technique. Compared with the hook plate, the stability of vertical and horizontal directions of the acromioclavicular joint was taken into account in the case of less bone trauma to avoid some complications caused by the rigid structure of the hook plate on the premise of ensuring the fixed strength similar to hook plate. This study retrospectively analyzed 43 patients with acromioclavicular joint dislocation admitted to Xi ‘an Honghui Hospital from January 2020 to January 2023. They were treated with the hook plate technique and allogeneic tendon coracoclavicular ligament reconstruction combined with the Kirschner wire technique. The two groups’ clinical data and postoperative efficacy were compared to explore the advantages and disadvantages of the two surgical methods.

## 2 Clinical data

### 2.1 Inclusion and exclusion criteria

Inclusion criteria: 1 Age ≥18 years; 2 X-ray and CT showed acromioclavicular joint dislocation; 3 Rockwood classification was type III and above; closed trauma, injury time ≤2 weeks; 5 The clinical manifestations were shoulder pain and limited activity after trauma. Exclusion criteria: 1 a history of shoulder surgery; 2 patients with severe medical diseases that can not tolerate anesthesia; 3 patients with incomplete clinical data; 4 combined with acromion, coracoid and other parts of the fracture; 5 patients with other surgical fixation methods. From January 2020 to January 2023,43 patients with Rockwood type III-V acromioclavicular joint dislocation were treated. Among them, 19 patients were treated with allogeneic tendon coracoclavicular ligament reconstruction combined with Kirschner wire fixation (allogeneic tendon group), and 24 patients were treated with hook plate (hook plate group). This study was approved by the Ethics Committee of Xi ‘an Red Cross Hospital. The patients and their families knew the surgical plan and signed the informed consent.

### 2.2 Surgical methods

#### 2.2.1 HP group

The patient took the beach chair position and raised the shoulder of the affected side. An arc-shaped surgical incision was taken at the acromioclavicular joint, and the distal end of the clavicle was separated and exposed to the acromioclavicular joint. The acromioclavicular joint was reset, and the hook plate was shaped correctly. The hook was inserted under the acromion to maintain the hook plate and the clavicle to attach as much as possible, and then the screw was fixed.

#### 2.2.2 AT group

**FIGURE 1:**
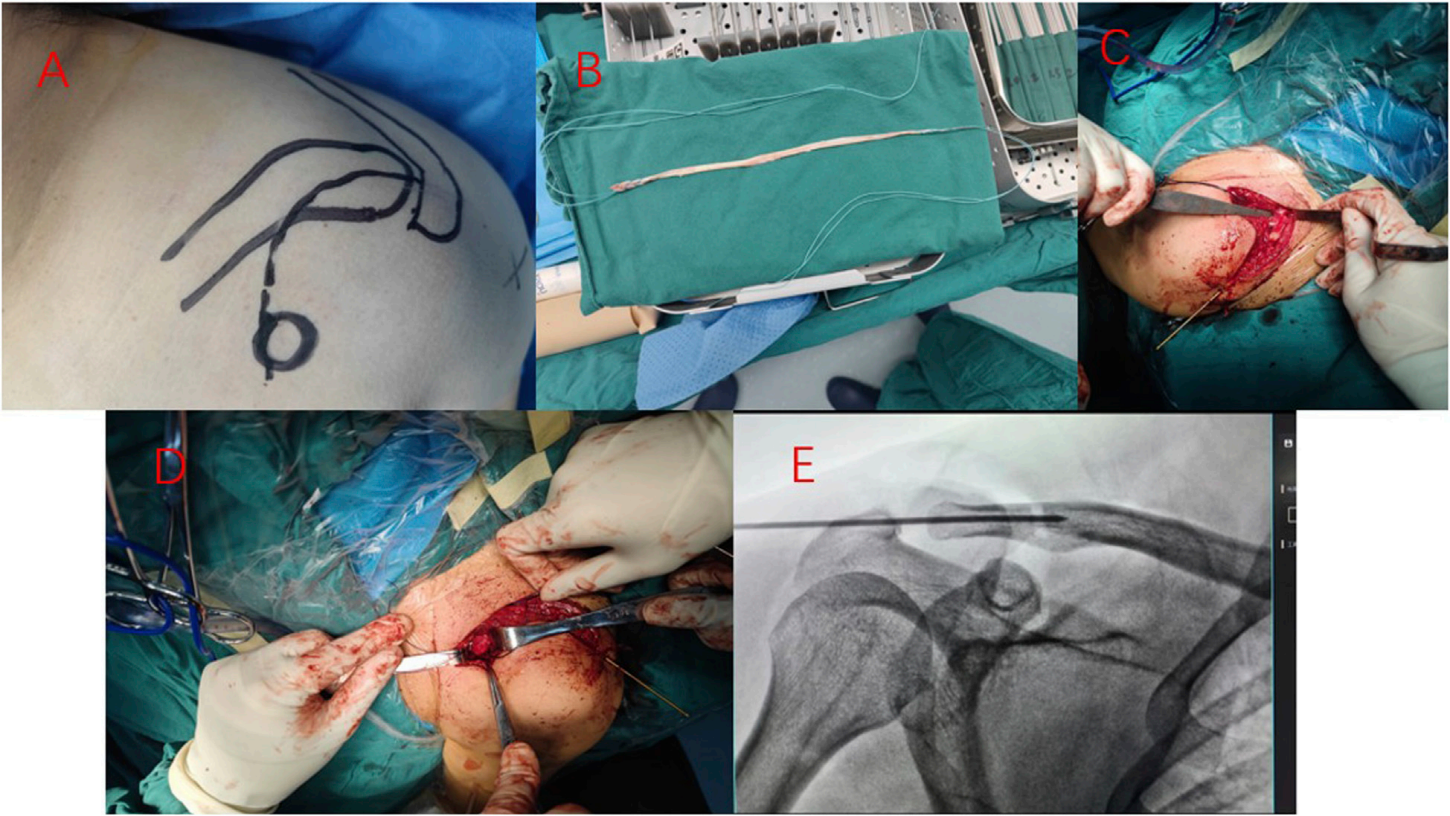
The steps of reconstruction of coracoclavicular ligament with allogeneic tendon combined with Kirschner wire fixation for acromioclavicular joint dislocation are shown in Figure 1. Surgical plan planning **(A)**, allogeneic tendon weaving **(B)**, drilling at the insertion of clavicular coracoclavicular ligament **(C)**, allogeneic tendon implantation **(D)**, intraoperative fluoroscopy of acromioclavicular joint **(E)**.

The patient took the beach chair position and raised the shoulder of the affected side. An arc-shaped surgical incision was taken between the acromioclavicular joint and the coracoid process to separate and expose the acromioclavicular joint and the coracoid process. The curved forceps guided the allogeneic tendon to cross the coracoid process below the coracoid process, reset the acromioclavicular joint, and percutaneously penetrated a Kirschner wire from the acromion to the clavicle. A hole was drilled at the attachment point of the clavicle coracoid process, and the allogeneic tendon bypassing the coracoid process was inserted. After tightening the tendon, the knot was sutured, and the position of the acromioclavicular joint was verified by fluoroscopy again, as shown in Figure 1.

### 2.3 Postoperative treatment

Symptomatic treatment was given after the operation, and the affected limb was fixed. According to the situation, K-wires in the tendon allograft reconstruction group were removed around 4–6 weeks, Shoulder abduction, flexion and external rotation exercises were performed in the first 8 weeks after operation. After 8 weeks, a more extensive range of functional exercises of the shoulder joint were performed step by step. The hook plate group underwent internal fixation removal 6–8 months after operation.

### 2.4 Observation indicators

The operation time, intraoperative blood loss, visual analogue scale (VAS) before operation and 2 weeks after operation were compared between the allogeneic tendon reconstruction group and the hook plate group (Focsa et al., 2023). Constant-Murley shoulder function score was performed 6 months after the operation, and the range of motion (ROM) of shoulder abduction and elevation was measured at the last follow-up. An X-ray examination was performed on the second day after the operation. The X-ray of the affected shoulder was regularly evaluated, and the recovery of shoulder function was recorded every month after the operation. Constant-Murley’s shoulder score included pain, activities of daily living, muscle strength, and range of motion, a total of 100 points. The higher the score, the better the shoulder function (Metzlaff et al., 2016).

### 2.5 Statistical analysis

SPSS 22.0 software was used for data analysis. Measurement data conforming to normal distribution and homogeneity of variance were expressed as mean ± standard deviation, and two independent samples t-tests were used to compare groups. Count data were compared using the χ2 or Fisher’s exact test, and *p* < 0.05 was considered statistically significant.

## 3 Results

**FIGURE 2:**
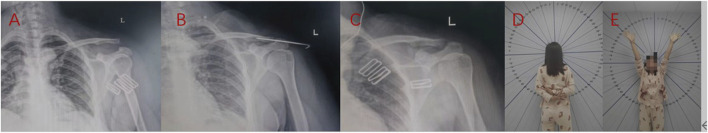
The reconstruction of acromioclavicular joint dislocation using allogeneic tendon coracoclavicular ligament showed that X-ray **(A)** before the operation, X-ray **(B)** on the second day after the operation, X-ray **(C)** after removal of Kirschner wire 6 weeks after the operation, and shoulder joint function **(D,E)** 1 year after the operation.

**FIGURE 3:**
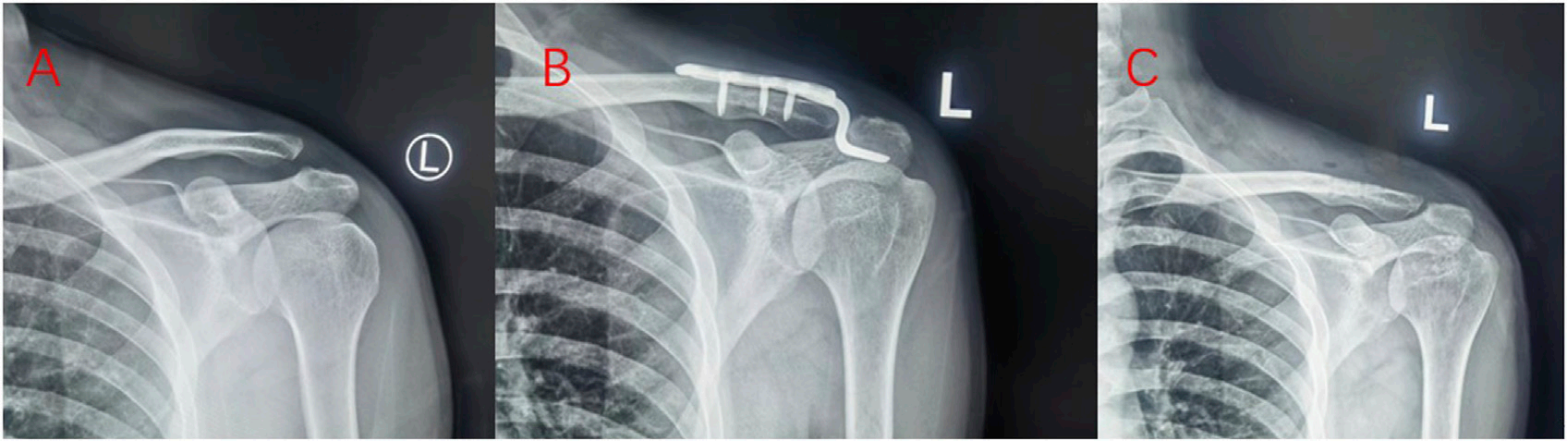
The acromioclavicular joint dislocation was fixed with the hook plate. Preoperative X-ray **(A)**, X-ray on the second day after operation **(B)**, and removal of hook plate X-ray **(C)** 8 months after operation.

All 43 cases were followed up. Rockwood classification was type III, IV, and V, as shown in Table 1. The mean follow-up time was 9.7 (9–12) months. During the follow-up period, there were no complications, such as infection, internal fixation failure, or secondary dislocation of the acromioclavicular joint. In the allogeneic tendon reconstruction group, there was 1 case (5.3%) of exudation around the Kirschner wire track. Considering the possibility of rejection, the Kirschner wire was removed at the fourth week. There were 5 cases (20.8%) of complications in the hook plate group, including 1 case of internal fixation stimulation, 2 cases of acromion impingement syndrome, 1 case of acromioclavicular joint osteoarthritis, and 1 case of shoulder joint stiffness. The complication rate of the allogeneic tendon group was lower than that of the hook plate group. Typical cases are shown in Figure 2 and Figure 3.

**TABLE 1:** Rockwood classification of acromioclavicular joint dislocation in two groups of patients.

Rockwood classification		AT group (n = 19)		HP group (n = 24)	
Type Ⅲ		11		14	
Type Ⅳ		2		3	
Type Ⅴ		6		7	

The intraoperative blood loss in the allogeneic tendon group was less than that in the hook plate group, and the difference was statistically significant (*p* < 0.001). The Constant-Murley shoulder function score of the allogeneic tendon group was higher than that of the hook plate group at 6 months after operation, and the difference was statistically significant (*p* < 0.001). The abduction and lifting activity of the allogeneic tendon group was greater than that of the hook plate group, and the difference was statistically significant (*p* < 0.001). At 3 days after operation, the VAS score of the allogeneic tendon group was lower than that of the hook plate group, and the difference was statistically significant (*p* < 0.001). At 14 days after operation, the VAS score of the allogeneic tendon group was lower than that of the hook plate group, and the difference was statistically significant (*p* < 0.001).

There was no significant difference in preoperative VAS score between the allogeneic tendon group and the hook plate group (*p* = 0.208). There was no significant difference in the operation time between the allogeneic tendon group and the hook plate group (*p* = 0.082), as shown in Table 2.

**TABLE 2:** Comparison of perioperative data and follow-up data between the two groups of patients.

Index	AT group (n = 19)	HP group (n = 24)	*p*-value
Operation time (min)	65.5 ± 4.2	67.7 ± 3.7 0.082	0.082
Intraoperative blood loss (mL)	33.5 ± 2.1	70.6 ± 4.7 < 0.001	< 0.001
Shoulder function score (points)	92.7 ± 2.1	81.6 ± 2.4 < 0.001	< 0.001
Shoulder abduction lifting activity (degree)	133.5 ± 3.0	115.1 ± 2.7 < 0.001	< 0.001
VAS score (points)			
Pre operatively	6.4 ± 0.8	6.1 ± 0.7 0.208	0.208
3 days after surgery	4.2 ± 0.9	7.0 ± 0.8 < 0.001	< 0.001
14 days after operation	3.1 ± 0.9	4.3 ± 0.8 < 0.001	< 0.001

## 4 Discussion

In the anatomy of the acromioclavicular joint, the acromioclavicular ligament plays a role in maintaining the stability of the anterior and posterior acromioclavicular joint. In contrast, the coracoclavicular joint maintains the stability in the vertical direction. When the ligament is broken, it will lead to the acromioclavicular joint dislocation. In clinical work, the conservative treatment effect of Rockwood type III and above acromioclavicular joint dislocation is poor, and surgical treatment is needed to correct the dislocation. The most widely used treatment for acromioclavicular joint dislocation is surgical fixation with the hook plate, which has good stability after fixation. However, hook plate fixation belongs to rigid fixation. Some scholars have found that non-rigid fixation is more in line with the biomechanics of acromioclavicular joints, which is conducive to anatomical reduction and later functional rehabilitation exercise. More and more scholars have used non-rigid fixation to treat acromioclavicular joint dislocation and achieved good clinical results (Mardani-Kivi et al., 2013; Pérez et al., 2023). The rigid fixed structure of the hook plate also makes the shoulder wear the acromion when the shoulder is undergoing abduction exercise rehabilitation, resulting in acromion impingement syndrome (Ko et al., 2023). Or secondary shoulder stiffness and many other complications, so it is usually necessary to remove the hook plate by secondary surgery (Lin et al., 2014; Chang et al., 2019). Currently, for treating acromioclavicular joint dislocation of Rockwood type III and above, the concept of reconstructing the anatomical structure of acromioclavicular ligament and coracoclavicular ligament has been paid more and more attention. Many related surgical methods include single-bundle, double-bundle and three-bundle ligament reconstruction. The stability of single-bundle reconstruction is difficult to guarantee. Double-bundle reconstruction is aimed at reconstructing the acromioclavicular ligament and coracoclavicular ligament, which are the most important in maintaining the stability of the acromioclavicular joint. Three-bundle reconstruction can increase the strength of the acromioclavicular joint after reduction and disperse the fixed stress based on double-bundle reconstruction (Park et al., 2018). The clinical treatment effect of double-bundle and three-bundle reconstruction is better, and its core purpose is to restore the stability of the anterior and posterior directions and vertical direction of the acromioclavicular joint. Simone‘s study used allogeneic tendon combined with screw to treat acromioclavicular joint dislocation. The surgical technique is similar to the fixed material properties used in this study, but the surgical method is different. In this study, only a single bone tunnel is drilled on the side of the clavicle. The allogeneic tendon passes through the bone tunnel and surrounds the coracoid process to complete the reconstruction of the coracoclavicular ligament. The single bone tunnel not only has less trauma, but also reduces the risk of clavicle fracture due to bone destruction compared with the double bone tunnel (Cerciello et al., 2021). In addition, scholar Yoon-Min innovatively used Mersilene band instead of autologous tendon to reconstruct the coracoclavicular ligament to obtain better clinical results. By wrapping the Mersilene band around the clavicle and coracoid process, the clavicle and coracoid process are gathered to complete the reconstruction of the coracoclavicular ligament. Obviously, the surgical technique is different from this study. Because the ends and the Mersilene band are limited to a certain range, the shallow grooves on the one side of the clavicle limit the activity of the Mersilene band. The stability of its fixation is worrying, if the shoulder joint is greatly rehabilitated or accepts a certain external force. Whether the position of Mersilene band will be lost when the reconstruction material is not broken, resulting in the failure of ligament reconstruction is obviously a certain biological safety hazard (Lee et al., 2022). The biomechanical study of the acromioclavicular joint shows that the ligaments that maintain the stability of the acromioclavicular joint mainly include the coracoclavicular ligament, acromioclavicular ligament and coracoacromial ligament, among which the coracoclavicular ligament has the most significant influence on the stability of the acromioclavicular joint (Tauber et al., 2016). The surgical method used in this study focusesfocuses on the coracoclavicular ligament combined with Kirschner wire fixation and elastic fixation and reconstruction of the coracoclavicular ligament to achieve a stable reduction and reconstruction of clinical treatment.

The clavicular hook plate changes the axial activity of the acromioclavicular joint and limits the micromotion of the acromioclavicular joint. Therefore, the range of motion of the shoulder joint is limited after operation, which affects the recovery of shoulder joint mobility. The clavicular hook plate fixation only achieves reduction and temporary high-strength stability on the anatomical alignment of the acromioclavicular joint. It does not solve the problem of local ligament rupture. When the clavicular hook plate is removed, the ligament of the acromioclavicular joint is completely healed. The stability of the acromioclavicular ligament may only rely on partial ligament healing and adhesion of the surrounding scar tissue so that the removal will reduce the stability of the acromioclavicular joint. A risk factor analysis of acromioclavicular joint reduction loss after hook plate fixation of acromioclavicular joint dislocation showed that 38 of 118 patients had reduction loss, with a loss rate of 32.20% (Lee et al., 2023). The hook plane angle and acromioclavicular arthritis are the risk factors for the loss of reduction after removing hook plate internal fixation for acromioclavicular joint dislocation. When the hook plane angle is too large, the hook end of the plate cannot be attached to the lower edge of the acromion, which leads to acromioclavicular osteolysis and acromioclavicular fracture.

The results of this study showed that the Constant-Murley shoulder function score at 6 months and the abduction and lifting activity of the shoulder joint at the last follow-up in the allogeneic tendon group were more significant than those in the hook plate group, and the difference was statistically significant (*p* < 0.001). The intraoperative blood loss, VAS score 3 days after operation and VAS score 14 days after operation in the allogeneic tendon group were lower than those in the hook plate group. It can be concluded that the allogeneic tendon group has less trauma, less intraoperative blood loss, and less early postoperative pain than the hook plate group, which is conducive to early rehabilitation functional exercise. The shoulder joint function score and shoulder joint mobility in the allogeneic tendon group were better than those in the hook plate group, which was beneficial for patients to return to everyday life and work as soon as possible. In terms of complication rate, there were 1 case in the allogeneic tendon group, with a complication rate of 5.3%, and 5 cases in the hook plate group, with a complication rate of 20.8%. The allogeneic tendon group had a lower complication rate. This may be related to the allogeneic tendon group. While ensuring an excellent acromioclavicular relationship reconstruction effect, because the coracoid process is not drilled in the clavicle drill single hole, the technique surrounding the coracoid process makes the local trauma of the bone tissue small. Then, the early pain is lighter, and a less rigid structure is implanted so that the flexibility of the acromioclavicular joint is not limited, thus obtaining a better clinical treatment effect.

By comparing the clinical data of the two fixation materials, it can be concluded that the reconstruction of allogeneic tendon combined with Kirschner wire fixation is more minimally invasive than the fixation of hook plate titanium alloy material, and the postoperative VAS score is lower. The difference is significant, indicating that the patients have less pain caused by surgical treatment. The shoulder function score at 6 months after operation was higher, and the difference was significant, indicating that allogeneic tendon reconstruction can achieve better surgical treatment effect. In summary, the effect of allogeneic tendon combined with Kirschner wire fixation in treating Rockwood III ∼ V acromioclavicular joint dislocation is better than that of clavicular hook plate. The patients have less early postoperative pain, better shoulder joint function and shoulder joint mobility, and a lower incidence of complications. This fixation technique is worthy of further clinical promotion. However, some things could be improved in this study. The patient’s shoulder Kirschner wire needs to be fixed for 4–6 weeks, and the needle track must still be cared for after the wound is removed. In addition, the sample size of this study is small, and the statistical results obtained may be biased. In the later stage, it is still necessary to expand the sample size to obtain statistical data that can more accurately reflect the clinical treatment effect.

## Data Availability

The original contributions presented in the study are included in the article/Supplementary material, further inquiries can be directed to the corresponding author.
